# Entrepreneurial intentions of Gen Z university students and entrepreneurial constraints in Bangladesh

**DOI:** 10.1186/s13731-023-00279-y

**Published:** 2023-03-09

**Authors:** Mohammad Imtiaz Hossain, Mosab I. Tabash, May Ling Siow, Tze San Ong, Suhaib Anagreh

**Affiliations:** 1 Faculty of Management, Multimedia University, Persiaran Multimedia, Selangor 63100 Cyberjaya, Malaysia; 2 College of Business, Al Ain University, Al Ain, UAE; 3 Faculty of Design and Architecture, Universiti Putra Malaysia, 43400 Serdang, Selangor Malaysia; 4 School of Business and Economics, Universiti Putra Malaysia, 43400 Serdang, Selangor Malaysia; 5 Department of Business Administration, Daffodil International University, Daffodil Smart City, 1207 Dhaka, Bangladesh; 6 Higher Colleges of Technology, Dubai, UAE

**Keywords:** Entrepreneurship, Attitude, Subjective norms, Behavioural control, Resilience, Constraints, Gen Z

## Abstract

This research examines a variety of restrictions preventing Bangladeshi youth, particularly Generation Z university students, from becoming involved in entrepreneurship. Moreover, the study examines the influence of Entrepreneurial Attitude (EA), Subjective Entrepreneurial Norms (SEN), Entrepreneurial Perceived Behavioural Control (EPBC), and Entrepreneurial Resilience (ER) on Entrepreneurial Intention (EI) of Bangladeshi Gen Z university students. A systematic literature review methodology following PRISMA procedure was performed to identify the relevant articles. A quantitative method with a positivism philosophy, cross-sectional time horizon and deductive approach was applied to the study. The data of 206 university students from the BBA department of ten universities were collected using convenience sampling and a self-administrated structured questionnaire survey. SPSS 26.0 and Smart PLS 3.0 were used to analyse the data. The output shows a positive and significant association amongst EA, SEN, EPBC, ER, and EI. Various constraints were identified from the literature and ranked based on the respondents’ feedback. This research will help entrepreneurs, scholars, policymakers and practitioners to build the entrepreneurial ecosystem and develop young people’s understanding of the entrepreneurial decision process and the importance of ER. This paper contributes through empirical investigation to an understanding of the actions that prevent Gen Z students from entrepreneurial activities; decisions are affected by socio-psychological constructions integrating ER with the Theory of Planned behaviour (TPB) model. Triple, Quadruple and Quintuple Helix models are considered supporting theories in this study to shed light on tackling the constraints. To the best knowledge of the researcher, integrating ER with TPB model’s constructs is a pioneer scholarly contribution in the context of South-East Asian, specifically Bangladeshi Gen Z students.

## Introduction

Bangladesh is a densely populated country with a high proportion of young people: according to the UN (2018), more than 50% of the 166.7 million people are under 24. Transformation into a developed country largely depends on Generation Z (i.e. below 27 years old in 2022). Almost 3.6 million people will remain unemployed in 2022 (ILO, 2022). The unemployment rate in 2021 is 5.23%, whereas it was 4.4% in 2019 (Statista, 2022). The youth unemployment rate was 14.7% in 2021, almost triple the national unemployment rate (World Bank, 2022).

**Fig. 1:**
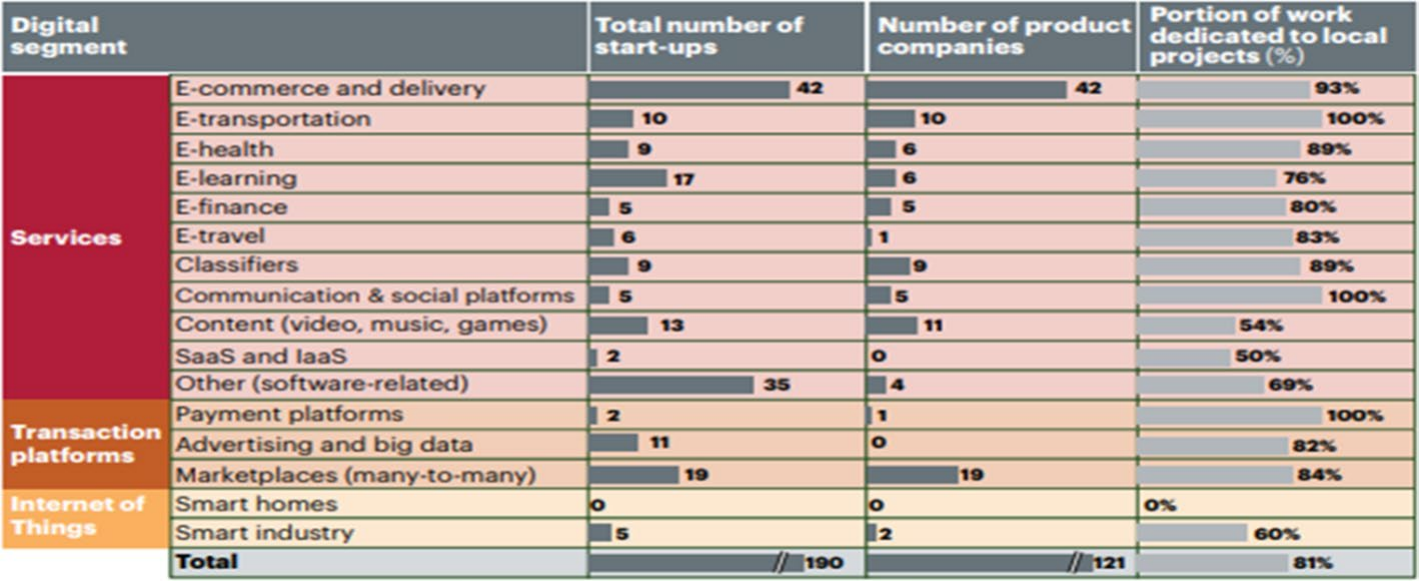
Types of Start-ups in Bangladesh (Kearney, 2017)

More than 800 IT and ITES (Information Technology enabled Services) companies are registered in Bangladesh, with a total approximate turnover of $200 million. More than 70% of these companies provide customised application services. Every year, 200 start-up companies enter the local business landscape. Although there were 1000 digital start-ups by 2016, about 600 have yet to progress beyond the idea stage (Kearney, 2017). Most provide e-commerce, e-transport, e-health, e-travel, e-payment platforms and e-marketing services (Fig. 1).

In Bangladesh, 125,000 small and 800,000 cottage industries have created job opportunities for 3.8 million people, according to a Financial Express report (2018); about 70% of these businesses are managed by the young. In addition, there are 1 million SMEs throughout the country, although 80% lack young entrepreneurs. Government policies have long stressed self-employment to provide opportunities for new entrants into the labour force (Hytii & Gorman, 2004). To stimulate economic growth, the Government of Bangladesh is emphasising new venture creation, women’s entrepreneurship and the advancement of SMEs. Despite the significant contribution of start-ups and government support, the failure rate of these ventures in Bangladesh is very high. As of 2019, the interest rate on bank loans is very high (10–12%), and the fixed deposit rate (9.75%) is similarly very high compared with neighbouring countries. Because of this opportunity cost of using savings as capital, businesses can rarely make only 10–15% profit daily (Kearney, 2017). Hence, investors are reluctant to support new businesses rather than place savings in FDR. Exploration of these and other constraints is an objective of this study.

Young people invariably choose business activities as the opportunities for conventional careers are few. One of Bangladesh’s biggest challenges is to reduce the 33% rate of youth unemployment. According to Hossain et al. (2018), this would improve the GDP, provide direct economic benefits for society, and reduce violence, crime and personal vulnerability. It also offers young people a sense of membership and opportunities to realise their dreams. Chigunta (2002) believes that independent employment leads young people to economic mainstreaming. Besides addressing socio-psychological problems and criminal activities resulting from unemployment, the study emphasises developing new expertise, encouraging creativity and flexibility, revitalising the local society through providing valuable goods and services, and making the new economic landscape accessible to young people.

Entrepreneurial activities can create jobs (Kritikos, 2014; Moses et al., 2016), drive the economy and develop the lifestyle, besides offering economic independence to young people in emerging nations such as Bangladesh (Ogunlana, 2018). Entrepreneurial activity is also an innovative way of generating income, self-reliability, livelihood and self-confidence (Maxwell, 2002).

However, entrepreneurial activity does not appear overnight (Zamrudi & Yulianti, 2020). It involves the interaction of cognitive processes and individuals’ behavioural attitudes to social, economic and cultural factors. Previous studies confirm that those with a strong Entrepreneurial Intention (EI) have high potential in entrepreneurship (Amofah & Saladrigues, 2022; Muslima et al., 2019; Jakopec et al., 2013). In the current research, we examined how the cognitive condition of a potential entrepreneur is directed by Entrepreneurial Attitude (EA), Subjective Entrepreneurial Norms (SEN) and Entrepreneurial Perceived Behavioral Regulation (EPBC) towards the intention to initiate a business venture. Higher intentions generate more entrepreneurial practices (Botsaris & Vamvaka, 2016). Prior studies have indicated that intentions are the single most influential determinant of actual behaviour (Ajzen & Fishbein, 1977; Souitaris et al., 2007), which is why it is imperative to inspect this cognitive condition. To explain the psychological aspects of entrepreneurship, the Theory of Planned Behaviour (TPB) is the most widely accepted model.

However, during this unprecedented period of volatility, uncertainty, complexity, and ambiguity (VUCA), adaptability and decision-making capacity are necessary to sustain entrepreneurship, demanding the incorporation of Entrepreneurial Resilience (ER). Therefore, we incorporate ER within this framework to provide a more detailed understanding of entrepreneurial behaviour. As a result, the current investigation contributes to the TPB studies by analysing an untested adaptive structural model in a domain that enhances the scope of the TPB model.

At the same time, the current COVID-19 pandemic is causing economic instability, creating uncertainty in the entrepreneurs’ mindset. Thus, it is crucial to understand how entrepreneurs survive in instability and what fuels entrepreneurship during difficult times. In this sense, ER can play a vital role. Individuals who exhibit resilience have a better chance of entrepreneurial success (Yang & Danes, 2015).

Fatoki (2018) defines ER as a dynamic adaptation process that helps entrepreneurs remain forward-looking despite the adverse market environment and constant exacerbating occurrences. Owing to their success in challenging environments, their direct knowledge of adversity and the informal organisational settings in which they work, entrepreneurs tend to be personally resilient and, therefore, resilient in business. Recent research illustrates considerable resilience amongst SMEs in extreme events; for example, in the aftermath of the earthquakes in Christchurch in 2010, the lack of written crisis management strategies did not affect business resilience (Battisti & Deakins, 2012). The Triple Helix perspective can be essential in tackling any unpleasant situation and ensuring a sustainable, innovative entrepreneurial culture. This analytical model was developed by Etzkowitz and Leydesdorff (1995, 2000) that established synergy amongst institutions: universities, industries and government agencies. Later on, Carayannis and Campbell (2009, 2010, 2011, and 2014) extended the model by including a fourth helix representing culture-based public, civil society and arts-based innovation and formed the Quadruple helix model. Aggarwal and Sindakis (2022) illustrates the triple helix concept and the quadruple helix innovation model. Another perspective added to the natural environments of society and developed the Quintuple Helix model. As a developing country, Bangladesh faces numerous challenges in employing the Helix models in the local entrepreneurial ecosystem and is still in the infancy stage. A few notable constraints include a lack of initiative from respective authorities, an outdated curriculum, insufficient industry support, less proactivity to develop entrepreneurial incubators, and policy-level barriers (Islam et al., 2020; Shabnaz & Islam, 2021).

On the other hand, researchers have sometimes specifically included resilience in the EI model. Krueger et al. (2000) contributed significantly to the development of the EI model, but they should have recognised the significance of the ER dimension. The research focus on resilience is minimal, primarily as the concept is comparatively new. In view of the above, a new integrated model has been established in the TPB framework, which, to the best knowledge of the researcher, has yet to be discussed in the previous literature. In order to confirm a consistent supply of entrepreneurs, scholars and practitioners, it is necessary to understand what factors promote or constrain entrepreneurship.

EI research has been conducted in other countries, including Italy (Campanella et al., 2013), Algeria (Izzrech et al., 2013), China (Kaijun & Sholihah, 2015), Malaysia (Muslim et al., 2019), Indonesia (Mangundjaya, 2009), Pakistan (Batool et al., 2015), Nigeria (Osakede et al., 2017), Ghana (Biney, 2019), Zimbabwe (Ndofirepi, 2020), Hungary (Perpék et al., 2021), Croatia (Bilić et al., 2021), Colombia (Campo-Ternera et al., 2022) and Spain (Martínez-González et al., 2019). In addition, several studies on Bangladeshi entrepreneurs have aimed to determine their background and psychological attributes as well as the barriers they face as entrepreneurs (Chowdhury, 2017; Nawaser et al., 2011; Uddin et al., 2015), although mainly focused on Gen Y (i.e. those born between 1977 and 1995 and now above 25 years of age).

As social norms evolve, motivations for being an entrepreneur can differ according to the cultural setting and the context of different generations (Shook & Bratianu, 2010). As Elfving et al. (2009) have argued, entrepreneurs received plenty more support from society than EU countries in societies like the United States. This is highly relevant for a developing country such as Bangladesh, where other resource constraints exist. Moreover, the characteristics of western countries that are more pro-market will be different in countries where the government more dominantly controls the economic system in its economic activities. Thus, the application of Helix models also varies based on the cultural and institutional context (Momeni et al., 2019). The diversity of behaviours between Gen Y and Gen Z and variation in cultures has led us to carry out this research; a few studies have addressed the EI and ER of Bangladeshi Gen Z. It is also important to research EA, SEN, EPBC, ER and EI in a variety of geographic contexts with various population cohorts since numerous sociological studies show a growing homogenisation of cognitive, affective-relative and behavioural trends resulting from globalisation (Nowak et al., 2006). This is relevant to younger generations, particularly Gen Z.

In sum, research and solid findings on recognising start-up impediments alongside examining the effect of EA, SEN, EPBC and ER on the EI of Gen Z, particularly concerning Bangladeshi university students, still need to be more sparse. This study is an attempt to fill this gap. Thus, the objectives of this paper are (a) to investigate the relationship between EA, SEN, EPBC and ER on the EI of Bangladeshi Gen Z students; and (b) to identify the constraints faced by Bangladeshi Gen Z students in creating a new venture.

To do so, the paper proceeds along the following lines. First, we reviewed the literature on EA, SEN, EPBC, ER, and EI, focusing on ER. From this review, we then derived four hypotheses tested with empirical data collected from 206 students randomly chosen from ten universities in Dhaka, the capital of Bangladesh. Dhaka is the national focal point of economic activity and a central education hub with 68 universities, setting the scope of the study. The quantitative data were analysed using SPSS and Smart-PLS software, with statistical methods well established in social science to put our hypotheses to rigorous testing. The methods applied are introduced, and the empirical findings are discussed. Finally, the practical and theoretical implications, future research directions, and the study’s conclusions are presented.

## Literature review

### Literature review methodology

**Fig. 2:**
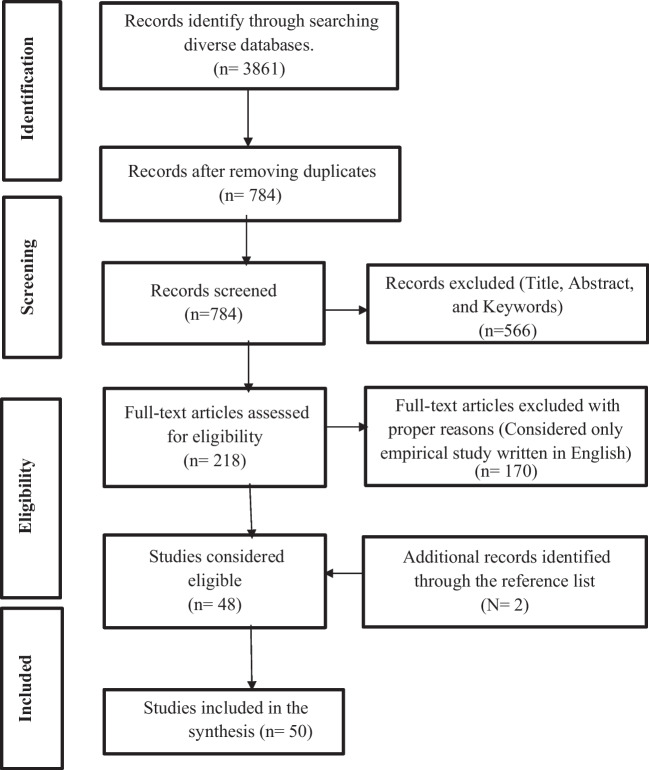
Article selection process. Source: Adapted from Moher et al. (2009)

The current study followed the PRISMA framework for reviewing existing literature. As mentioned in the PRISMA guidelines, the scoping procedure was used to extract the most relevant articles on youth university students' entrepreneurial intention and relevant factors. This practice facilitated regulating the critical lessons' obligatory features and classifying possible search keywords. The PRISMA model of the current study was adapted from Moher et al. (2009), as illustrated in Fig. 2, which consisted of four steps named identification, screening, eligibility and included. The procedure started with identifying the literature and was followed by data screening and an eligibility test; the steps were concluded by including the article for conducting the study.

### Identification and screening

The identification process of the articles has been conducted through the key search items, ‘(entrepreneurial intention) AND (university students*) AND (Attitude and youth and entrepreneurial intention)’ AND (Attitude and youth entrepreneurship and entrepreneurial resilience) (AND, OR acknowledged as Boolean operators) have been used in Web of Science, Scopus, Google scholar, Emerald, Science Direct. As the Web of Science is recognised as one of the top websites for identifying articles, it was utilised first. It provides subscription-based access to multiple databases containing extensive articles for different academic disciplines. Secondly, the Scopus database was utilised as it includes more journal categories than the Web of Science. In addition, Emerald and Science Direct are used as they allow the authors to filter the result by the heading of articles, the name of authors, the year of publication, the category of articles, and/or location, and a new search can be initiated within the results. The abstract, references and full text are presented along with the title of each article, and they can be accessed. Keywords are highlighted at the time of going through the abstracts. The results can be narrowed down by the heading of articles, the name of authors, the year of publication, the category of articles, location, and a new search can be started within the results.

These search strategies identified 3861 records in total. Only those articles were considered for the current review study, which was published between 2011 and 2021, and the final identification of the article was performed in March 2021. Table 1 indicates the protocols that were followed for the identification and screening of articles. After conducting the article identification, the duplicates were removed, and the number of articles was narrowed down to 784.

**Table 1:** Protocols followed for the identification and screening of articles

Items	Description
Keywords	Entrepreneurial intention, university students, youth entrepreneurship, attitude, youth and entrepreneurial resilience
Boolean operators	‘AND’ between keywords; OR between database search fields
Search fields	Title, Abstract, Keywords
Time	2011–2021

The article selection criteria have been performed based on the exclusion and inclusion process. The exclusion and inclusion criteria of the articles are illustrated in Table 2.

**Table 2:** The exclusion and inclusion criteria of the articles

Inclusion criteria	Exclusion criteria
Papers that are published in the period 2011–2021	Book chapters, books, working papers, or other types of non-peer-reviewed publications
Papers are available in reputed journals	Articles that are not written using the English language
Papers that have provided information regarding entrepreneurial intention, university students, attitude, youth and entrepreneurial resilience	Articles that represent reviews or surveys regarding previous work

As stated earlier, 3861 articles were identified by following several protocols mentioned in Table 1. After removing the duplicate from the total number of identified articles were narrowed down, the number of unique articles was found 784. Later on, all the articles were screened based on their title, abstract, and keywords mentioned in the articles, and 566 were excluded. The full articles were gone through in case of insufficiency and ambiguousness of the information in the abstract (Stewart et al., 2015). The full text of the rest 218 articles was assessed and screened again based on the title, abstract, keywords, language and the types of articles.

In contrast, the second screening process excluded 170 articles. Only empirical and peer-reviewed articles were considered for the study as they will assist in maintaining the quality of the review, as mentioned by Moher et al. (2009). After completing the screening process total of 48 articles were considered eligible for the study and read many times to gather important information. Later on, 2 more articles from the reference list from those 48 articles were added to the 48 articles. Finally, 50 quality articles (Appendix A) were considered key for the literature review and justifications in other sections.

## Theoretical and empirical background

### Generation Z

Randstad (2014) and Hoque et al. (2018) describe Gen Z as the post-millennials, the social media generation, digital natives, dotcom-children, the digital—generation, and the generation born after 1993. The McCrindle Research Centre (2018) positions the average age of Gen Z in the late teens. Bangladesh will be home to some 85 million young people in 2020, of which there are around 85 million (UN, 2018). They are called technologically advanced, mature early, pampered, empowered and all over the Network (Ensari, 2017). It is challenging for the government to achieve sustainable development without the entrepreneurial endeavours of this vast section of the population. With this in mind, the government intends to build a youth-favoured entrepreneurship environment.

### Theory of planned behaviour (TPB)

This study used Ajzen’s Theory of Planned Behaviour (TPB) as an underpinning theory introduced in 1991. TPB is the most persuasive and widely used socio-psychological model for elucidating and predicting human behaviour in different contexts. To understand behavioural change, researchers have applied TPB in diverse settings. This framework helps to understand the behaviour of entrepreneurs. TPB is an extended version of the Theory of Reasoned Action (TRA), predicting intention more accurately at a specific time and place. It consists of three independent antecedents, Attitude towards the behaviour, Subjective norms and Perceived behavioural control. Moreover, Triple Helix, Quadruple, and Quintuple Helix models are shading light to develop the resiliency and sustainability of entrepreneurs fostering innovativeness.

### Entrepreneurial intention (EI)

Bandura (1997) said that intention precedes a particular activity or anticipates results in a specific impending condition. Ozaralli and Rivenburgh (2016) claimed that intention act as a direct antecedent of real behaviour, and the stronger the intention for behaviour, the bigger the success of behaviour prediction or actual behaviour. In entrepreneurship, Oguntimehin and Olaniran (2017) stated that EI is people’s inclination to perform entrepreneurial behaviour and engage in entrepreneurial activities, be self-employed and build a start-up business. Individuals must have an entrepreneurial inclination to be an entrepreneur (Polas et al., 2019). Mohan (2022) highlighted the positive aspects of EI, stating that persons with EI are more likely to recognise economic opportunities than persons not interested in entrepreneurship. Due to the positive outcomes associated with entrepreneurial activity, researchers and policymakers alike are motivated to acquire an in-depth knowledge of EI (Amofah & Saladrigues, 2022).

Scholars have confirmed that EI is a legitimate factor. Numerous studies show that it provides substantial opportunities for the researcher to understand the entrepreneurial process and envisage entrepreneurial activities by recognising EI’s antecedents (Farrukh et al., 2017). Generally, it echoes something already existing, started deliberately and not inadvertently (Nabi et al., 2017). Previous studies stated that university students have sufficient knowledge and training to choose their career path. Students’ intention to be entrepreneurs is high, at 30.4% (Sandri, 2016). Researchers such as Jena (2020), Souitaris et al., (2007) support this view highlighting the importance of entrepreneurial education to develop EI. However, Colette et al. (2005) disagreed and mentioned that teaching could propel entrepreneurial motivation. Nevertheless, it is a daunting task as many procedures, including making firm decisions (Tiwari et al., 2017). Socio-demographic factors also significantly influence building EI (Polas et al., 2019).

Koe et al. (2012) revealed the determinants of EI amongst the millennial generation by drawing on TPB. Unambiguously, they established the relationship between knowledge, experience and ties, attitude, social norms, perceived behavioural control and personality traits towards entrepreneurial tendency. Ismail et al. (2009) demonstrated Malaysian undergraduates’ EI by examining the relationship between the Big-Five personality factors, contextual factors and EI. Ryan and Deci (2000) stated that motivation is fundamental to being an entrepreneur, and it involves energy, direction, perseverance and intention and is the core of biological, psychological and social regulation. There is a link between motivation, intention and behaviour (Amofah & Saladrigues, 2022).

Motivation drives us into action. Edelman et al. (2010) assert that inspiration may be the catalyst to turn a latent intention into entrepreneurship. To understand entrepreneurial motivation, we first study the individual’s behaviour and psychological intention, and TPB helps to realise these factors.

### Entrepreneurial attitude (EA)

Attitude towards behaviour refers to a person’s opinion, judgement and assessment of oneself towards that behaviour (Ajzen, 1991). This belief comes from personal experience, available information sources and the influence of others. For example, someone may be positive toward entrepreneurship because his parents have a business. This attitude produces favourable and unfavourable attitudes and positive and negative outcomes. Chowdhury (2017) noted that the intrinsic need of the person to do something unique, something in a relevant field, has been established as a major aspect. This comprises entrepreneurial motivation, efficiency and capacity. Passion and commitment to power are essential. For Aktaruddin (1999), personal attributes are key to entrepreneurial success or failure. Ajzen (2002) suggested that this attitude can be evaluated by attitude (valuable vs worthless) and experiential quality (pleasant vs unpleasant). Ferriera et al. (2007) concluded that a person’s attitude positively affects intention. Iskandarini (2014) explained that attitudes affect behaviour through effects on intention. Intention depends on the situation and personality.

EA considers sovereignty, jeopardy, work, remuneration and other benefits, while entrepreneurial aptitude includes opportunity identification, viability screening, and creative problem-solving skills (Fitzsimmons et al., 2005). Chuah et al. (2016) find that attitude towards behaviour has the most significant impact on EI amongst university students in Malaysia. A study conducted in Algeria by Mohammed et al. (2017) supports this finding. This study will investigate the influence of Entrepreneurial Attitude (EA) on Entrepreneurial Intention (EI). The hypothesis is formulated as follows:

***H1:*** There is a positive and significant relationship between EA and EI

### Subjective entrepreneurial norms (SEN)

SEN is called the normative belief of entrepreneurs. It refers to perceived social pressure to engage or not engage in entrepreneurship's target behaviour or activities (Francis et al., 2004). Opinions of others, especially parents, friends, relatives, experts and many more, shape this belief, playing a significant role and pressuring a person to perform a particular behaviour (Yean, 2015). To measure these norms, both injunction and descriptive norms can be used. Injunction norms refer to what others think “ought to be done”. Descriptive models do not consider what others think but “what others do normally”. Khuong and An (2016) found that the subjective norms of Vietnamese students did not influence EI. Najafabadi et al. (2016) also documented similar results with Iranian students. Conversely, Yang (2013) found a positive relationship in the Chinese students' context. Therefore, the following hypothesis is formulated:

***H2:*** There is a positive and significant relationship between SEN and EI

### Entrepreneurial perceived behavioural control (EPBC)

EPBC refers to an entrepreneur’s belief in their capabilities to perform certain behaviours (Brouwer et al., 2009). It differs based on the situation and action. It can be used to predict behavioural achievement. The study outlines both internal controls (such as personality) and external restrictions (such as opportunities) (Ajzen, 1991). This behaviour substantially impacts individuals’ performance (Yean, 2015). Real behaviour depends on both stimuli and insight into the complexity of behaviour (Solesvik et al., 2012). Although few researchers mixed with PBC and self-efficacy, Ajzen (2002) specifies that it is a wider construct since it encompasses and perceived controllability of the behaviour. Nguyen (2017) found a positive relationship between EPBC and EI, although Mohammed et al. (2017) found an insignificant relationship. Hence, we hypothesise:

***H3:*** There is a positive and significant relationship between EPBC and EI

### Entrepreneurial resilience (ER)

Tonis (2015) and Fletcher et al. (2013) first used this term in psychology, and now it is widely applied in multi-disciplinary research. ER is the aptitude of the entrepreneur to handle severe personal and market circumstances and disruptive events (Fatoki, 2018). Resilient entrepreneurs view adverse situations positively besides resolving an unstable and challenging marketplace (Morisse & Ingram, 2016). Due to their performance in difficult situations, their intimate experience of challenges, and the informal organisational contexts in which they function, entrepreneurs are often highly resilient and can apply this to business. Entrepreneurs sometimes make wrong decisions or misjudgements because the information needs to be more precise and complete. To avoid those pitfalls by creating a knowledge-based entrepreneurial environment, institutional collaboration, training, and guidance are essential where Quadruple Helix and the Quintuple Helix models can provide multidimensionality (Morawska‑Jancelewicz, 2021) and applicable as a flag, a guiding heuristic (Cai & Etzkowitz, 2020). Entrepreneurs must therefore keep up-to-date knowledge of evolving contingencies by adapting their priorities and strategies (Adeniran & Johnston, 2012; Bullough & Renko, 2013).

Previous studies focus on whether more resilient entrepreneurs will succeed in their businesses (Ayala & Manzano, 2014) and how they adapt to changes and recovery under harsh conditions (Bullough & Renko, 2013). Aude D’Andria et al. (2018) found that strong resilience contributes to the success of business acquisitions. Reinmoeller et al. (2005) and Fisher et al. (2016) also found a positive link between entrepreneurs’ resilience and the growth of their companies. Ofunoye (2017) found an insignificant relationship between resilience and business success, estimated by employee numbers, profitability growth and sales growth. Given that entrepreneurs are exposed to a high risk of failure and uncertainty, this study aims to assess the relationship between ER and EI.

***H4:*** There is a positive and significant relationship between ER and EI.

### Conceptual framework

**Fig. 3:**
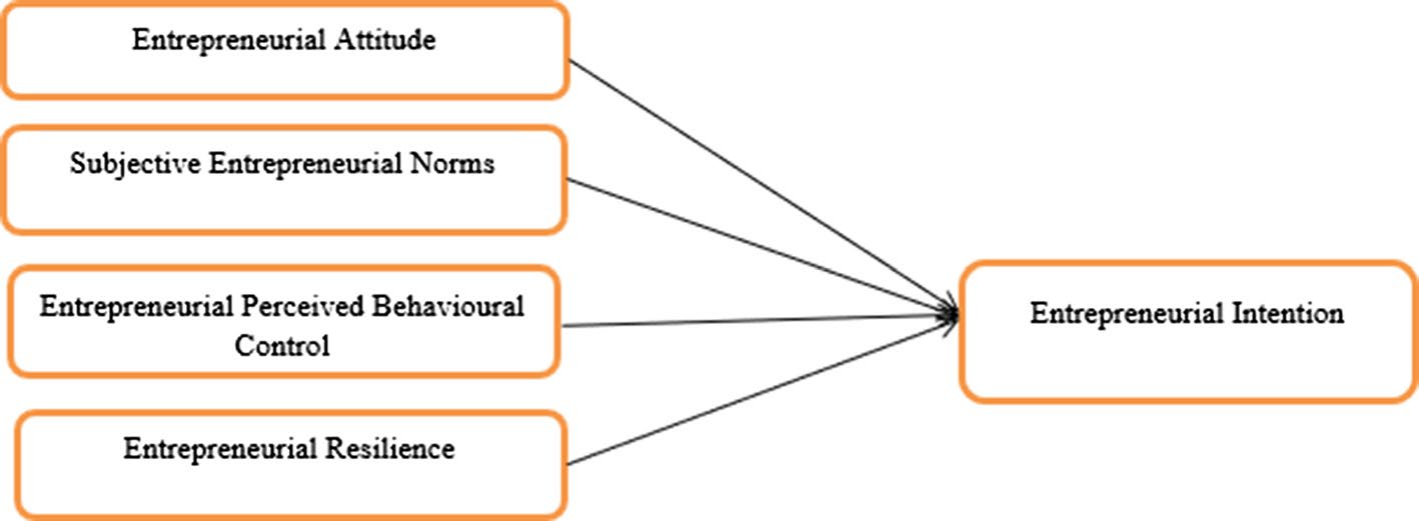
Conceptual framework (adapted from Ajzan, 1991)

The conceptual framework shown in Fig. 3 is adapted from Ajzan’s (1991) TPB and the literature review.

## Methods

### Research philosophy and design

Research philosophy refers to identifying the nature of the study, for which different techniques of pragmatism, realism, and positivism are present (Ying et al., 2021). The study’s research design contributes to defining the entire strategy selected for integrating several parts of the research to flow correctly, which helps in effectively identifying the problem addressed in the study (Ying et al., 2021).

Since the study aims to assess the impact of EA, SEN, EPBC and ER on EI empirically and systematically, thus, the positivism philosophy, quantitative research methodology, cross-sectional time horizon and deductive research approach are appropriate. A Positivist worldview allows researchers to discover practical information using a hypothetical deductive observation process. Since the data were collected in a single time frame, this study is cross-sectional. As this study used theories to describe variables, develop hypotheses and test empirically, it follows a deductive approach. Considering the nature of the study and to validate the model by obtaining and analysing the data, a quantitative method was applied. The essence of this analysis is a causal study that explains the relationship of cause and effect between the independent and dependent variables. This study employs a survey technique because it collects a good amount of data from the population cost-effectively and generalises findings typical of the entire population. The questionnaire is an appropriate tool to achieve the research objectives as it is economical, time-saving, and covers the research area widely. It provides quantifiable data for analysis. Three academics and three students checked the questionnaire and a pilot study with 30 participants to ensure the content and face validity.

### Population and sample

The researcher contacted five public universities (University of Dhaka, Jahangirnagar University, Jagannath University, Bangladesh University of Textiles, Sher-e-Bangla Agricultural University) and five private universities (North South University, BRAC University, Independent University, East West University, Daffodil International University) situated in Dhaka. The questionnaires were distributed to the final year university students of the BBA department conveniently and psychically as they are potential entrepreneurs. The university students aged 18–25 were only considered for this study to ensure the participation of the Gen Z generation. A filter question regarding age was applied in the questionnaire to identify the specific respondents. Selecting appropriate respondents is crucial as it impacts the findings. Convenience sampling was used as it is a widely used tool in entrepreneurship studies (Amofah & Saladrigues, 2022; Fayolle et al., 2006). Dhaka was chosen because 32.8% of the population aged 15 and above lives in this city (BBS, 2018), and it is the focal point of all business activities. Two hundred twenty-five responses were collected. After reviewing the data to confirm the validity of the analysis, 19 responses were found to be invalid for statistical analysis due to incomplete data, leaving a total of 206. Hair et al. (2019) state that the findings of SEM are sensitive to the sample size and propose that SEM research should present a minimum of 100 and a maximum of 200 cases.

Additionally, a priori power analysis was performed using G*Power 3.1.9.4 software (Faul et al., 2007) to determine the sample size required for the proposed model. The power analysis results indicated a minimum of 129 sample size to achieve 95% statistical power for a medium effect (0.15) at a significance level of 5% (0.05) for the proposed structural model based on four predictors. Thus, the number of responses achieved is adequate.

### Data analysis procedures

SPSS and Smart PLS 3.0 were used to analyse the data. After the actual data gathering, the raw data are examined through SPSS version 26.0, and then finally, Smart-PLS 3.0 is utilised. Demographic data were analysed by conducting frequency analysis through SPSS. Non-response bias was ensured by comparing the mean and standard deviation of the first 30 early responses and the last 30 late responses following Wallace and Cooke’s (1990) procedure. Smart-PLS evaluate the consistency of the measurement model or the external model in terms of its reliability and validity. Correlation analysis was performed. The relationships between latent variables in the conceptual framework direction coefficient (β) and determination coefficients (R2) are shown in the structural model or internal model. The regression coefficient (β) tests all hypothetical paths in the system. The value of β is tested using the PLS Bootstrap technique to observe the proposed hypotheses in the structural model.

### Questionnaire development and measurement of constructs

***Demographic information:*** The first part of the questionnaire includes demographic information (gender, age, marital status, monthly income). The following embedded the constructs from TPB: attitude, subjective norms, and perceived behavioural control. The items are:

***EA****:* Keen to take advantage of new business opportunities, positive outlook on business failure, willing to take the risk (Utami, 2017), satisfied with entrepreneurship (Mohammed et al., 2017).

***SEN****:* Confident role of the family, the support of friends, colleagues’ appreciation (Mohammed et al., 2017), career advisors, and teachers have a positive impact (Schoof, 2006).

***EPBC:*** Leadership may determine success, having confidence in the ability to manage the business (Utami, 2017), preparedness to start, optimistic about the business's success (Mohammed et al., 2017).

***ER:*** Capable of adapting to change, seeing the humorous side of challenges, dealing with stress will improve me, bouncing back from illness or difficulty, achieving goals despite difficulties, remaining concentrated under pressure, not easily discouraged by disappointment, thinking of self as a strong person, managing negative feelings (Fatoki, 2018).

***EI:*** Firm determination about the start-up, goal-oriented (Mohammed et al., 2017), choosing a career as a better option, entrepreneurship education (Utami, 2017).

The final part of the questionnaire focuses on the constraints on entrepreneurship, with items adapted from Schoof (2006). All the items in the questionnaire were measured on a 5-point Likert scale, with scale responses varying between Disagree and Strongly Agree. A five-point Likert scale is used to ensure consistency between the variables and prevent misunderstanding amongst respondents (Ackfeldt & Coote, 2005). In addition, the five-point Likert-type scale is used to improve the response rate and response efficiency and reduce the “frustration level” of the respondents (Sachdev & Verma, 2004).

## Results and discussion

### Demographics analysis

As presented in Table 3, 80.58% of respondents were male; 6.80% were aged 18–19 years, 28.16% were 20–21, 35.92% were 22–23, and 29.13% were 24–25. In addition, 86.89% of respondents were single, and 13.11% were married. For monthly income, 21.36% earned US$100–200, 23.79% US$201–300 USD, 32.04% US$301–400, 16.99% US$401–500 and 5.83% US$501–600.

**Table 3:** Respondent’s demographic profile

Characteristics	Frequency	Percentage
Gender		
Male	166	80.58
Female	40	19.42
Age		
18–19 years	14	6.80
20–21 years	58	28.16
22–23 years	74	35.92
24–25 years	60	29.13
Marital status		
Single	179	86.89
Married	27	13.11
Divorced		
Separated		
Window		
Monthly income (US dollar)		
100–200	44	21.36
201–300	49	23.79
301–400	66	32.04
401–500	35	16.99
501–600	12	5.83
Total	206	100

The first step in evaluating the model is to test the measurement model in which Cronbach’s alpha and composite reliability are tested for reliability, and composite reliability and discriminant validity are tested for convergent and discriminant. Table 4 confirms that the AVE value of every variable is above 0.50; the CR and Cronbach’s Alpha value is above 0.70; factor loadings are above 0.60; all fall within the accepted range. EI is shown to have a significant effect (R square = 0.827 or 82.7%) on independent variables.

**Table 4:** Measurement of model assessment

Constructs	Items	Loading	AVE	CR	Alpha	R-Square
Entrepreneurial Attitude (EA)	EA1	0.853				
	EA2	0.884	0.696	0.872	0.785	
	EA3	0.761				
Subjective Entrepreneurial Norms (SEN)	SEN4	0.853				
	SEN5	0.843	0.73	0.891	0.815	
	SEN6	0.867				
Entrepreneurial Perceived Behavioural Control (EPBC)	EPBC7	0.793				
	EPBC8	0.912	0.694	0.872	0.779	
	EPBC9	0.79				
Entrepreneurial Resilience (ER)	ER10	0.873				
	ER11	0.836	0.744	0.897	0.829	
	ER12	0.877				
Entrepreneurial Intention (EI)	EI13	0.813				
	EI14	0.786	0.705	0.872	0.861	0.827
	EI15	0.872				
	EI16	0.883				

Source: Authors’ own work

Discriminant validity represents one construct’s real differentiation from other constructs. Table 5 demonstrates the evaluation of discriminant validity for assessing the model, following the criterion provided by Fornell and Larcker (1981). All variables’ square root of the AVE (in bold) describes the highest within a range of 0.833–0.862. Thus, discriminant validity is confirmed between variables.

**Table 5:** Values of correlations between the LV and square roots of the AVE values in the main diagonal in the SEM

	Constructs	1	2	3	4	5
1	EA	**0.834**				
2	EI	0.616	**0.840**			
3	EPBC	0.687	0.678	**0.833**		
4	ER	0.687	0.777	0.665	**0.862**	
5	SEN	0.563	0.643	0.479	0.688	**0.854**

The diagonal is the square root of the AVE (in bold) of the latent variables and indicates the highest in any column or raw

Source: Authors’ own work

**Fig. 4:**
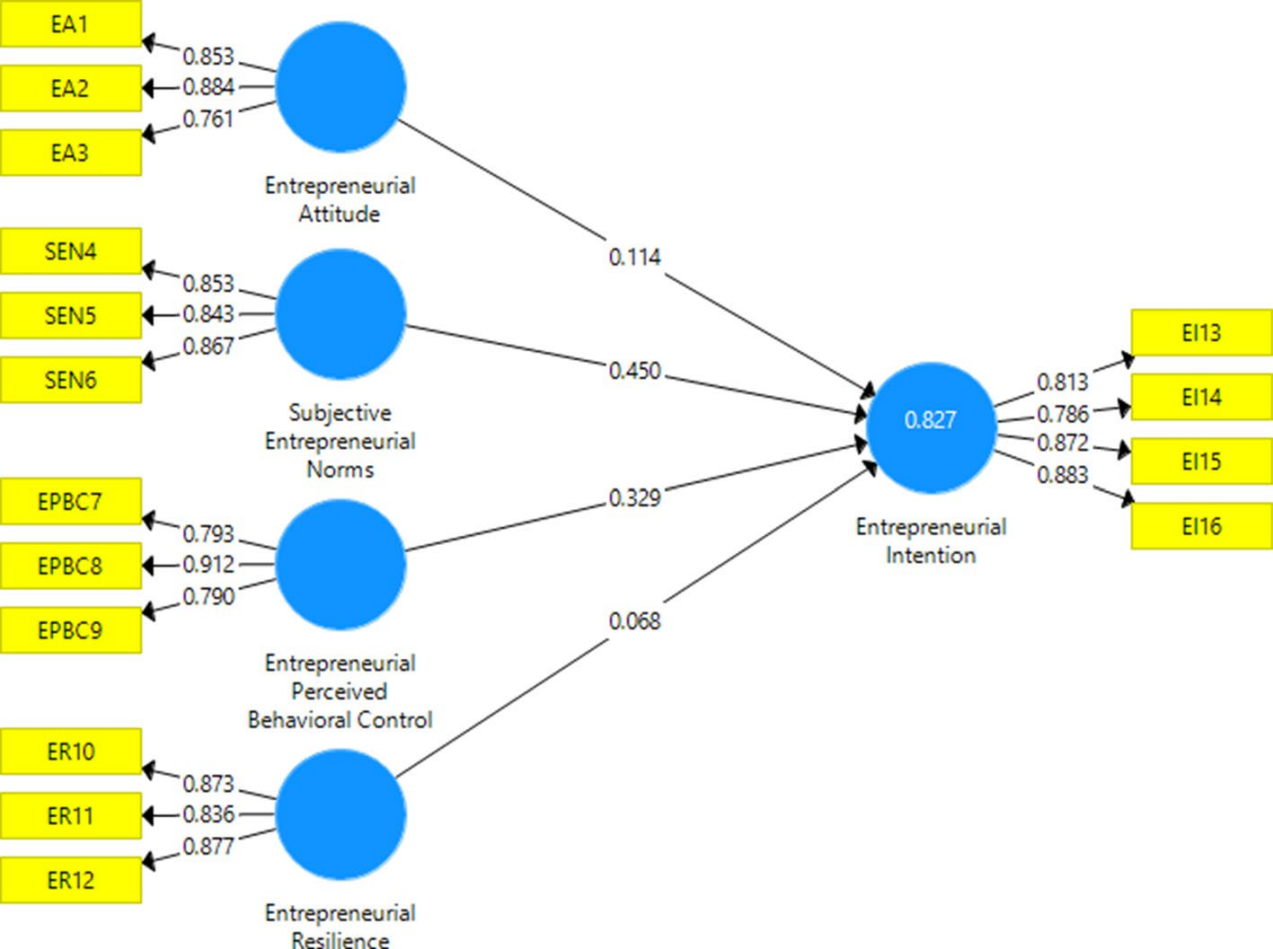
Standardised results of SEM calculations

The next stage is verifying the structural model's validity once the measurement model is in place. Figure 4 shows the structural model assessment. The bootstrapping process with a resample of 500 was also implemented to figure out the t-values and R square.

The bootstrapping procedure is performed to estimate t statistics and confidence intervals since PLS does not have any criteria for distribution assumption (Chin, 1998). The relationship between independent and dependent variables in the inner path model is observed, and the path estimation or hypothetical relationships are carried out. The regression coefficient (β) tests all hypothetical paths in the model. The value of β is tested using the PLS Bootstrap technique to observe the proposed hypotheses in the structural model.

Table 6 shows the results of the analysis for testing the hypotheses. A positive and significant connection between EA and EI (β = 0.692, *t* = 15.778, *p* < 0.001) supports hypothesis 1. As Bangladesh is an overpopulated country with many social problems, there is wide scope for starting a business to solve these problems. That is, the mindset of youth to be entrepreneurs is already present. This result is consistent with Ohammed et al. (2017), Halberstad et al. (2019) and Tarnanidis et al. (2019). It indicates that attitude is a variable that acts as the interface between values that concentrate on social norms and other subjective variables and specific behavioural and cognitive variables such as motivation and action. This finding is in dissonance with the study of Ridha et al. (2017), who found an insignificant influence on attitude towards entrepreneurship.

**Table 6:** Result of direct and indirect effect hypotheses

Hypothesis	Relationship	Std. beta	Std. error	*t*-value	*p*-value	Decision
H1	EA → EI	0.692	0.044	15.778	0.000	Supported
H2	SEN → EI	0.703	0.046	15.299	0.001	Supported
H3	EPBC → EI	0.693	0.042	16.468	0.000	Supported
H4	ER → EI	0.743	0.043	17.108	0.001	Supported

For the second hypothesis, a positive and strong relationship is found between SEN and EI (β = 0.703, *t* = 15.299, *p* < 0.001), supporting hypothesis 2. Bangladesh has a collective culture, which strongly affects social life. Social influences encourage individuals to believe life will improve if they succeed in business. As a result, more and more young people are venturing into business. This association is supported by Mirjana et al. (2018) and Yang (2013), although Ohanu and Shodipe (2021) and Shook and Bratianu (2010) found the contrary result, that peer norms do not affect EI. A possible justification for this result may be the nature of the relatively collectivist community. In a collective society, young people are dependent on their parents. However, our result does not support this view, perhaps because of growing individualism in families, with young people becoming more self-centred and motivated to aspire to a free career path. Undoubtedly, the causal relationship between SEN and EI is significant, considering that intention is the variable that best predicts EA and affects goal setting and other tasks to be performed in building an enterprise (Kim & Jang, 2018).

For the third hypothesis, the results indicate a positive and significant relationship between EPBC and EI (β = 0.693, *t* = 16.468, *p* < 0.001). Thus, hypothesis 3 is acknowledged. Bangladesh is a vibrant country full of energy. The continuous growth of the SME sector and the booming industry of start-ups encourage a firm belief in the success of entrepreneurship careers. This reflects young people’s behavioural control, which is well embedded within the entrepreneurial ecosystem. This outcome is well supported by Nguyen (2017). EPBC concerns how well people regulate their behaviour. These values, as well as mobilising and maintaining coping mechanisms, minimise stress and anxiety. Previously, research has shown that individuals with greater power use higher coping strategies (Leana & Feldman, 1994). Conversely, Ohanu and Shodipe (2021) and Ridha et al. (2017) found an insignificant association between EPBC and the EI of young agricultural entrepreneurs.

Finally, we found a positive and significant connection between ER and EI (β = 0.743, *t* = 17.108, *p* < 0.001), supporting hypothesis 4. This exciting finding means that the higher the resilience of entrepreneurs, the greater they will be EI, even with a small value. Individuals take lessons from their surroundings and bitter experience. There is a view that availability of the resources (human resources, financial resources and tools and equipment) develops the entrepreneurial attitude (Ohanu & Shodipe, 2021). Problems such as the unavailability of resources can make the person mentally stronger and determined to find new ways of succeeding. In Fatoki (2018), entrepreneurship was correlated with attitudes towards freedom and risk-taking. An entrepreneur with a deep desire to be one succeeds because he or she can overcome constraints.

Our results are associated with TPB, which indicates that resilience can counteract the adverse effects of stressful situations. An optimistic attitude and the ability to respond positively are essential psychological tools that can help with stress tolerance and promote coping, personal well-being and adaptation to challenging situations (Joseph & Linley, 2008). Although ER operates within the system, it is not a fixed characteristic. Instead, resilience works more at the base level and can be affected by epigenetic influences from the external world—negatively from adversity, for example, or favourably with support from mentors. This research contributes to the literature on entrepreneurship and resilience by exploring a comprehensive and robust EI model.

A descriptive analysis is conducted to achieve the abovementioned objective (b) to identify the constraints faced by Bangladeshi Gen Z students in creating a new venture. The key constraints are ranked based on the analysis considering the mean score. This identification will assist stakeholders in determining the priority and focus. Table 7 shows the key constraints: predominantly lacks the skill, with a mean score of 4.45 out of 5. Training, incubation initiative, and coordinated government efforts are Inadequate. Most training curricula need to be updated and more practical. Tailor-made training and advice with technical aspects need to be improved. Training institutions and the business community need to be connected. Entrepreneurs needed to receive correct guidance about what to do initially and when facing difficult feedback from reality. The respondents also expressed concern over Bangladesh’s political unrest, which ranked second (4.29). This country experienced numerous political protests and hunger strikes, causing communication problems, delaying product delivery, and adding additional costs. Problems with the infrastructure include frequent power shutdowns, load shedding, an unstructured transport system, and so on. While these two problems are not directly related to Gen Z but rather are national crises, they can have more negative effects on the young than on adults.

**Table 7:** Key constraints

Constraints	*N*	Rank	Mean	Std. Deviation
Financial risk	206	9	4.0900	0.82993
Social risk	206	12	3.8200	0.83339
Lack of capital	206	4	4.2500	0.90314
Lack of skill	206	1	4.4400	0.68638
Administrative hurdles	206	6	4.1400	0.71095
Gender discrimination	206	13	2.9110	1.19844
Workload	206	14	2.7800	1.29162
Corruption	206	7	4.1300	0.86053
Competition	206	5	4.1900	0.72048
Market demand	206	3	4.2700	0.72272
Lack of family support	206	11	4.0200	1.18048
Infrastructural problem	206	10	4.0300	0.89279
Political unrest	206	2	4.2800	0.86550
Lack of technology	206	8	4.1010	0.88192

### Theoretical implications

From a theoretical perspective, the study provides a more comprehensive and robust approach than Ajzen’s (1989) TPB model by integrating ER with the TPB constructs. ER is a crucial factor that can assist entrepreneurs withstanding internal and external shocks. This factor was rarely explored in the entrepreneurship domain, and its effect on entrepreneurial intention and success has been inconclusive (Gismera Tierno et al., 2021; Fatoki, 2018). The findings of the study can fill the void. Including ER with TPB develops a novel theoretical model, and the study's empirical findings can be a valuable contribution to the entrepreneurship domain. We reserve Quadruple Helix and the Quintuple Helix model constructs for our further study; however, the discussion of these models in the current study will help the diverse stakeholders to realise the importance of close collaboration amongst academia industry government as well as democracy and ecology (Carayannis et al., 2020, 2021) for developing EI and ER. Previous researchers applied Quadruple Helix and the Quintuple Helix models to developing academic entrepreneurship (Samo & Huda, 2019), social innovation (Morawska‑Jancelewicz, 2021), business model creation (Shin, 2014), entrepreneurial university (Feola et al., 2021; Puangpronpitag, 2019; Yildiz, 2021) and regional entrepreneurship (Li et al., 2020; Sá et al., 2018). Assessing the emerged constraints in the shed of helix models can provide valuable insights for developing countries and other geographies.

### Managerial implications

Since the young people of Bangladesh regard a positive outlook towards all the variables described in this study and it is determined that this cohort has a favourable attitude towards entrepreneurship. This will assist the stakeholders in entrepreneurial development in educational institutions, government agencies and private organisations to provide appropriate stimuli. Therefore, policymakers can use the results of this research to develop policies to enhance the environmental capacity for sustainable entrepreneurial development in their regions. The findings will help them achieve their transformation objectives towards a more competitive local economy. Explicitly, the responses indicate that there is scope for change in this area to boost the impact of the entrepreneur in the teaching and learning process.

The results would help small businesses to meet their goals of upgrading their local economy into a more efficient one. The responses show that there is potential for improvement in political stability, financial capital support and skill enhancement to promote entrepreneurship and innovation. Sindakis and Aggarwal (2022) and Sitaridis and Kitsios (2019) also suggested providing quality support, skills and resources for better youth entrepreneurship.

The study findings will provide small businesses with strategies to enhance emergency response by undertaking training and entrepreneurship seminars, specifically regarding resources, pressure, transition, failure management and coping mechanisms. The study lets relevant government bodies like the SME Foundation of Bangladesh learn more about Helix models, TPB and young people’s ER and how it impacts their personal and business lives. This training will help to establish more robust resilience preparation for young entrepreneurs. The importance of resilience in the mind of entrepreneurs is crucial for the future pandemic. Entrepreneurship educators can use the model implemented in this study as a quantitative method to define the degree to which the model variables promote or inhibit the resilience of university students and, more widely, other sources of potential entrepreneurs. Managers and academics will better understand whatever needs to be developed. This framework could also provide diagnostic assistance for developing effective and efficient curricula and educational programmes aimed at creating entrepreneurial activities. Deliberate entrepreneurial practice, business management skills and input from colleagues and advisors are especially relevant to improving resilience (Lent & Brown, 1996).

### Policy implications

It is expected that the outcomes of the research will inspire policy debate on the issues that foster or impede young people’s entrepreneurship and the policy measures that may ease the entrepreneurial journey for Gen Z. This study can inspire corporates or angel investors to come forward and reshape their strategies and restructuring their commitment towards community and society. Those engaged in entrepreneurial development can set the priority, policies, and strategies from the study’s takeaway to develop resilience in entrepreneurs. Since installing an incubator and implementing a model requires investment, and our study also found capital inadequacy (Table 7) is a major issue, government, university research centres and financial institutions should consolidate their effort to tackle this challenge.

### Limitations and future research directions

Some limitations could be considered. A single cohesive set of undergraduate BBA students in Dhaka was studied to assess the entrepreneurial behavioural state and resilience. Although this leads to new avenues of investigation for this community, a more appropriate method is to research other department students at other universities or cities. Therefore, other sample populations should be included to generalise the results. Similar studies might also be conducted on different cohorts, for example, with the alums or mature entrepreneurs or a comparison amongst different generations. Analysing the data on an aggregate level may neglect potential statistical variations and heterogeneity of EIs and ER in different groups of demographic actors, especially those with an entrepreneurial family history.

Additionally, the study explored only five constructs using previously developed items, and thus, future studies should consider scale development by exploring themes from entrepreneurial psychology or other domains. Future research may use mediating or moderating variables such as smart technologies (big data, predictive analytics etc.) influence or application of knowledge management, cultural orientation, and ambidextrous behaviour to provide interesting insights. Integrating Helix models with TPB and ER can deliver new perspectives and potential topics to investigate in future.

## Conclusions and recommendations

This research aims to deliver an overview of youth entrepreneurship concentrating on actual barriers and spurs to Gen Z in particular, with an evaluation of relationships amongst EA, SEN, EPBC, ER and EI. This paper has empirically examined how the actions of the students of Gen Z towards entrepreneurial activities/decisions are affected by socio-psychological constructs. The positive and significant relationships amongst variables indicate that those factors are valid in the context of Bangladeshi Gen Z students. As Bangladesh is a collectivist society and the values inherent in a previous generation continue into the following, young people are primarily influenced by the traditional concepts of their parents, teachers, older siblings and elders, which may justify this result. Moreover, in the Bangladeshi context, we explored the essential constraints that hinder Gen Z from being successful entrepreneurs, identifying the factors of growing entrepreneurial intention to improve the situation.

New programmes must be developed by fostering academia-industry-government collaboration in different cultural and national environments to promote entrepreneurship amongst young people. Entrepreneurs should listen to and learn from their internal voices without imitating anyone. The best way to achieve this is to explore their strengths and weaknesses. Interpersonal skills such as critical thinking, financial literacy and people management are needed. At the same time, parents should not impose their wishes on their children.

In order to promote the development of entrepreneurship, the government and other financial institutions should provide bank lending to entrepreneurs at lower interest rates. Political parties are responsible for stabilising policy by not calling unnecessary strikes, protests, etc. In order to create a better business ecosystem, multinationals and private organisations should emerge.

Paying respect to the value of desirability from a subject’s point of view, universities should pay more attention to boosting student self-performance and collective productivity by implementing educational programmes with a focus on entrepreneurial development courses/techniques embedded in the curricula. Core business training is essential for successful and sustainable socio-economic development in educational and other institutions, including business faculty curricula, since enterprise training harmony and coordination amongst policy institutions is crucial.

## Data Availability

The data that support the findings of this study are available based on request.

## Appendix A

### Findings from the review

**Table:**

No	Author and year	Method	Theory	Results
1.	Amofah and Saladrigues (2022)	Quantitative	Theory of Planned Behaviour	-Did not find a significant relationship between Males and Females about their entrepreneurial intentions - Relationship between parental self-employment (PSE) and perceived behavioural control (PBC) is stronger for Males than Females
2.	Ayala and Manzano (2014)	Quantitative	Human capital theory	-Three dimensions of resilience (hardiness, resourcefulness and optimism) help to predict Entrepreneurial success - The key factor in predicting the success of the entrepreneur is resourcefulness - The influence of optimism on the success of their businesses is greater for women than for men
3.	Batool et al. (2015)	Quantitative	Theory of Planned Behaviour	-Personal control, self-esteem, and creativity with mediating role of self-efficacy were found to have significant and positive relationships with online self-employment intention -Achievement was found to have no significant relationship with online self-employment intention
4.	Bilić et al. (2021)	Qualitative	–	-High bureaucracy and administrative barriers act as inhibiting factors to further development of academic entrepreneurship
5.	Biney (2019)	Qualitative	–	Youth have plans to innovate and grow businesses, but the high cost of credit is a barrier
6.	Botsaris and Vamvaka (2016)	Quantitative	Theory of Planned Behaviour	-Attitude toward entrepreneurship is predicted by the expectation that entrepreneurship will be followed by a given outcome - Intrinsic rewards of entrepreneurship are stronger predictors of entrepreneurial attitude than extrinsic rewards and that the dimensions of attitude toward entrepreneurship exert a differential impact on entrepreneurial intention, with affective attitude appearing to be more strongly related to intention than instrumental attitude
7.	Bullough and Renko (2013)	Quantitative	–	Entrepreneurial self-efficacy–—defined as a belief in one’s ability to be an entrepreneur–—and resilience are particularly important
8.	Iskandarini (2014)	Quantitative	–	The effect of barriers towards entrepreneurial intentions for the workforce is positive, which means the higher resistance value, the higher the entrepreneurial intention of the community for entrepreneurship
9.	Campanella et al. (2013)	Quantitative	Human capital theory	Entrepreneurship is significantly associated with seven variables: type of education, academic internship experiences, achievement of a master’s degree, achievement of a PhD, international study experiences, family culture, and university infrastructure
10.	Campo-Ternera et al. (2022)	Quantitative	The systemic entrepreneurship theory Theory of Planned Behaviour	Great impact of potential entrepreneurial capacity on effective entrepreneurial capacity, determined by the direct effect of personal traits and life skills, the family as a moderating element, as well as the mediating role of entrepreneurship training processes
11.	Chuah et al. (2016)	Quantitative	Theory of Planned Behaviour	behavioural factors, namely attitude, subjective norm and perceived behavioural control, have significant effect on entrepreneurial intention It is also found that perceived barriers and perceived support have positive impact on attitude and subjective norms respectively
12.	d’Andria et al. (2018)	Qualitative	Theories of effectuation and causation Broaden-and-build theory of positive emotion	-In high uncertainty, strong entrepreneurial resilience and shift of logics of action can contribute to the success of a business takeover -This study identifies forms of resilience during the business takeover process that helped the entrepreneur overcome adversity and succeed
13.	Esfandiar et al. (2019)	Quantitative	Theory of trying Theory of planned behavior	-Desirability is the main determinant of entrepreneurial goal intention (EGI), followed by self-efficacy, feasibility, opportunity, attitude, and collective-efficacy, while social norms do not influence EGI -EGI strongly influences entrepreneurial implementation intention
14.	Farashah, (2013)	Quantitative	Entrepreneurial Potential Model Theory of planned behavior	-Completion of one entrepreneurship course increases the likelihood of having entrepreneurial intention by 1.3 times -Wald criteria demonstrate that fear of failure, desirability of entrepreneurial career, entrepreneurs’ status in society, self-efficacy and education and training are significant predictors of entrepreneurial intention. Perceived opportunity is not a strong but a moderate predictor
15.	Farrukh et al. (2017)	Quantitative	Social capital theory	Family background was found to have a positive impact on the EIs of students -A positive relationship between self-efficacy and EIs. Consciousness, extroversion and openness to experience are positively linked with EIs while neuroticism and agreeableness did not show any relationship
16.	Fatoki (2018)	Quantitative	The resiliency model The grounded theory of personal resilience The attribution theory	There is a significant positive relationship between entrepreneurial resilience and individual and organisational success
17.	Feola et al. (2021)	Quantitative	Triple helix model	Effectiveness of technology transfer initiatives and instruments adopted is not independent from the context in which universities operate and are involved
18.	Fisher et al. (2016)	Quantitative	–	Resilience in entrepreneurs comprises hardiness and persistence; that entrepreneurs are more resilient than other populations; and that resilience does predict entrepreneurial success
19.	Hoque (2018)	Quantitative	Resource Based Theory and Resource Advance Theory	EO and OC were significantly related to SME performance and OC was found to mediate the relationship between EO and SME performance
20.	Hossain et al. (2018)	Quantitative	–	Fresh graduates need to change their demanding attitude and at the same time, they must adopt more employability skills in order to get a job placement
21.	Islam et al. (2020)	Quantitative	–	-The lack of confidence and calmness in confronting difficulties, lack of aspiration to be a successful boss, lack of favorable environment for entrepreneurial activities, lack of encouragement and learning on entrepreneurship, and lack of capital and business knowledge are significant prohibiting factors for the business graduates to be entrepreneurial in Bangladesh
22.	Jakopec et al. (2013)	Quantitative	Theory of planned behavior Model of the entrepreneurial event	-self-assessment of entrepreneurial tendencies and abilities is positively associated with the perceptions of entrepreneurial self-efficacy and desirability of entrepreneurship, which also contributes positively to explanation of entrepreneurial intentions. In addition, it was found that entrepreneurial tendencies and abilities influence entrepreneurial intentions only indirectly, through entrepreneurial self-efficacy and desirability of entrepreneurship -Finally, entrepreneurial tendencies and abilities, entrepreneurial self-efficacy and desirability of entrepreneurship together, explain most of the variance of the entrepreneurial intentions
23.	Jena (2020)	Quantitative	Theory of planned behavior	The results showed a significant positive impact of attitude towards entrepreneurship education on entrepreneurial intention
24.	Kaijun and Sholihah (2015)	Quantitative	Theory of planned behavior	This study demonstrate the significance of subjective norm and perceived behavioral control to entrepreneurial education in Chinese students. Furthermore, this study also found indirect effect of perceived behavioral controls on entrepreneurial intention with entrepreneurial education as an intervening variable among Chinese students
25.	Khuong et al. (2016)	Quantitative	The entrepreneurial event theory Theory of planned behavior	-Prior entrepreneurial experience, external environment and perceived feasibility were the three independent variables that significantly affected the positive perception toward entrepreneurship and consequently, they had positively indirect effect on entrepreneurship intention. On the other hand, perceived feasibility and personal trait significantly affected the negative perception toward entrepreneurship and provided negatively indirect effect on the entrepreneurship intention
26.	Lee et al. (2015)	Quantitative	-	-Unemployment is associated with young adults' heavy episodic drinking and possibly cigarette use, but not cannabis use -Moreover, for all three substances, the detrimental impact of unemployment on substance use seems to be exacerbated among young adults who spent their childhood and adolescence in a lower SES household. Public health efforts that provide other viable and affordable options to cope with unemployment among young adults from low SES backgrounds are needed to address this disproportionate concentration of adverse impacts of unemployment on behavioral health
27.	Mangundjaya (2009)	Quantitative	Theory of planned behavior	-There was no significant correlation between Adversity Quotient and Entrepreneurial Intention amongst University students -However, there is a significant correlation between Resilience (Adversity Quotient) and Entrepreneurial Intention amongst employees
28.	Martínez-González et al. (2019)	Quantitative	Theory of planned behavior	-There are no significant differences in the responses to the items or in the causal relationships of the model between both countries (Spain and Poland) -This confirms the relevance of a homogenizing generational approach at a global level that allows the application of policies to promote the entrepreneurial intention for the entire segment
29.	Mirjana et al. (2018)	Quantitative	Theory of planned behavior	-Personal attitudes towards entrepreneurship, subjective norms and perceived behavioural control are positively related to one’s entrepreneurial intentions. The innovative cognitive style has also been found to be significant in creating one’s intention to become an entrepreneur
30.	Mohammed et al. (2017)	Quantitative	Theory of planned behavior	-Students’ attitudes towards entrepreneurship and subjective norms, have a significant effect on behavioral intentions to entrepreneurship. On the other hand, perceived behavioral control had no significant effect
31.	Ndofirepi (2020)	Quantitative	Theory of Planned Behaviour Bird’s Theory of Implementing Entrepreneurial Ideas Shapero and Sokol’s Theory of Entrepreneurial Event	-The effects of entrepreneurship education variable had a positive and statistically significant relationship with need for achievement, risk-taking propensity, internal locus of control and entrepreneurial goal intentions -Moreover, need for achievement, risk-taking propensity and internal locus of control accounted for a statistically significant amount of variance in entrepreneurial intentions -However, of the three psychological traits, only need for achievement partially mediated the relationship between the effects of entrepreneurship education and entrepreneurial goal intentions
32.	Nguyen (2017	Quantitative	Theory of planned behavior	-Attitude toward entrepreneurship and perceived behavior control are positively related to entrepreneurial intention - Subjective norm fails to generate a significant impact on entrepreneurial intention. Entrepreneurial intention is significantly influenced by two components of TPB model which are attitude toward entrepreneurship and perceived behavior control
33.	Oguntimehin and Olaniran, (2017)	Quantitative	Theory of planned behavior	Entrepreneurship Education significantly influences students’ entrepreneurial intentions
34.	Ohanu and Shodipe (2021)	Quantitative	Theory of planned behavior	The study showed the robustness of TPB model in the face of available resource that attitude, subjective norms and perceived behavioural control are behavioural factors that can be positively influence by several exogenous factors to enhance EIMW students’ entrepreneurial intentions
35.	Najafabadi et al. (2016)	Quantitative	Theory of planned behaviour Shapero's entrepreneurial event model	-The order of effect of latent variables on entrepreneurial intention was entrepreneurial skill, self-efficacy, attitude toward entrepreneurship, psychological traits, and social norms
36.	Osakede et al. (2017)	Quantitative	Human capital theory, Shapero and Sokol’s entrepreneurial event theory, Bandura’s theory of social learning and Ajzen’s theory of planned behaviour	subjective norm, perceived behavioural control and family business background significantly predicts students’ interest in entrepreneurship. Engagement in entrepreneurial activity has no significant effect on students’ academic performance. Findings suggest relatively low entrepreneurial engagement among students with significant differences across gender
37.	Polas et al. (2019)	Quantitative	Theory of planned behavior	-Significant relationship between educational degree specialization, nationality, gender, entrepreneur parent, child position, marital status, age towards the tendencyto become an entrepreneur -Moreover, the study further didn’t find any relationship between performance (CGPA), religion and abroad experience towards the tendencyto become an entrepreneurthan earlier findings
38.	Potocan et al. (2021)	Quantitative	Great person and psychological characteristics theories	-The empirical research revealed differences in Slovenian and Croatian students’ perception about (a) needed academic activities and (b) significance of the offered university activities, for the development of their entrepreneurial abilities -Additionally, the results reveal that the impact of students’ gender and study level on their perception about the importance of the offered academic activities is not significant for most of the considered activities
39.	Ridha et al. (2017)	Quantitative	Theory of planned behavior	Respondents have highly average trends on behaviour belief, normative belief, motivation to comply, control belief, control belief power and intention. While, only the evaluation of the consequence to give the medium trend is about 50.26 per cent
40.	Sitaridis and Kitsios (2019)	Quantitative	Motivation-opportunity-ability theory Theory of planned behavior	-Lack of entrepreneurial knowledge and skills have a major impact on EIs of students. On the contrary, self-motivation towards entrepreneurship acts as an antidote -Finally, the differences in the perception of barriers and motivation, between the two genders and role model groups, were also examined
41.	Shabnaz and Islam (2021)	Quantitative	Theory of planned behavior	-Autonomy and market opportunity has significant positive impact whereas barriers like financial and government support, lack of skills has significant negative impact on the student’s entrepreneurial intentions
42.	Shin (2014)	Quantitative	Triple helix model	-Entrepreneurs should focus their attention on combining SNS resources with smart device characteristics, helping them create value in a profitable way
43.	Solesvik et al. (2012)	Quantitative	Entrepreneurial event theory Theory of planned behavior	-Students reporting higher levels of perceived desirability, perceived feasibility, attitude toward the behaviour (i.e. enterprise) and perceived behavioural control were more likely to report the formation of entrepreneurial intentions -No significant negative interaction effect between perceived desirability and perceived feasibility was detected
44.	Gismera Tierno et al. (2021)	Quantitative	The Entrepreneurial Event Model	Perceived feasibility and perceived desirability have as determining factors of Entrepreneurial Intentions
45.	Tiwari et al. (2017)	Quantitative	Theory of planned behavior	Creativity showed a strongest positive relationship followed by emotional intelligence
46.	Uddin et al. (2015)	Quantitative	–	-Start-up challenge is neglecting knowledge-based innovation, being their own boss is the main motivation to engage in business, parents and family mainly influenced young people to start business while financial risk is the most pressing de-motivator to start-up business
47.	Yang (2013)	Quantitative	Theory of planned behavior	-Attitude represented the most effective predictor of entrepreneurial intention, followed by subjective norms, and then perceived behavioral control -Gender and parents' entrepreneurial experience had a significant impact on entrepreneurial attitude, subjective norms, perceived behavioral control, and entrepreneurial intention -Effective entrepreneurship education could significantly enhance perceived behavioral control and entrepreneurial intention
48.	Yang and Danes (2015)	–	Patterson’s Family Adjustment and Adaptation Response theory	Spousal commitment (negative) and business demand (curvilinear) were significantly associated with breakeven point. Entrepreneur’s business confidence (positive), life outlook (positive), and business demand (curvilinear) were associated with business success
49.	Yean et al. (2015)	Quantitative	Theory of planned behavior	-Attitude and subjective norms have positively influenced intention to return to work among respondents. Perceived behavioural control however, has a non-significant impact on respondents' intention to return to work
50.	Zamrudi and Yulianti (2020)	Quantitative	Theory of planned behavior	-Entrepreneurial self-efficacy shows non-significant results on entrepreneurial intention -However, the most considerable effect of the supporting condition demonstrated better results on entrepreneurial self-efficacy rather than on behavioural control

## Abbreviations

EA: Entrepreneurial attitude
SEN: Subjective entrepreneurial norms
EPBC: Entrepreneurial perceived behavioural control
ER: Entrepreneurial resilience
EI: Entrepreneurial intention
ITES: Information technology enabled service
